# Substance P and pain chronicity

**DOI:** 10.1007/s00441-018-2922-y

**Published:** 2018-10-03

**Authors:** W. Zieglgänsberger

**Affiliations:** Max Planck Institute of Psychiatry, Munich, Germany

**Keywords:** Substance P, Chronic pain, Inflammation, Neuroplasticity, Long-term storage

## Abstract

Substance P (SP) is a highly conserved member of the tachykinin peptide family that is widely expressed throughout the animal kingdom. The numerous members of the tachykinin peptide family are involved in a multitude of neuronal signaling pathways, mediating sensations and emotional responses (Steinhoff et al. in Physiol Rev 94:265–301, 2014). In contrast to receptors for classical transmitters, such as glutamate (Parsons et al. in Handb Exp Pharmacol 249–303, 2005), only a minority of neurons in certain brain areas express neurokinin receptors (NKRs) (Mantyh in J Clin Psychiatry 63:6–10, 2002). SP is also expressed by a variety of non-neuronal cell types such as microglia, as well as immune cells (Mashaghi et al. in Cell Mol Life Sci 73:4249–4264, 2016). SP is an 11-amino acid neuropeptide that preferentially activates the neurokinin-1 receptor (NK1R). It transmits nociceptive signals via primary afferent fibers to spinal and brainstem second-order neurons (Cao et al. in Nature 392:390–394, 1998). Compounds that inhibit SP’s action are being investigated as potential drugs to relieve pain. More recently, SP and NKR have gained attention for their role in complex psychiatric processes. It is a key goal in the field of pain research to understand mechanisms involved in the transition between acute pain and chronic pain. The influence of emotional and cognitive inputs and feedbacks from different brain areas makes pain not only a perception but an experience (Zieglgänsberger et al. in CNS Spectr 10:298–308, 2005; Trenkwaldner et al. Sleep Med 31:78–85, 2017). This review focuses on functional neuronal plasticity in spinal dorsal horn neurons as a major relay for nociceptive information.

## Substance P, a member of the tachykinin peptide family

### History

Substance P (SP) was first isolated from equine brain and gut (v Euler and Gaddum 1931) and later from bovine hypothalamic tissue and identified as an undecapeptide (Chang and Leeman 1970; Chang et al. 1971; Pickel et al. 1977). In mammals, the three main tachykinins are SP, neurokinin A (NKA) and neurokinin B (NKB). Tachykinin family members are ubiquitously distributed in all mammalian tissues and body fluids. In the nervous system, the highest densities of SP are found in the dorsal horn of the spinal cord, the substantia nigra and the amygdala (Ribeiro-da-Silva and Hökfelt 2000). The observation that SP is expressed in the dorsal root ganglia (DRG) and dorsal horn but not in the ventral horn was taken as evidence in favor of its involvement in primary afferent sensory neurotransmission (Lembeck 1953; Lembeck and Holzer 1979; Lembeck 2008; Pernow 1953).

### Tachykinin receptors, synthesis and release

The tachykinins derive from alternate processing of three *TAC* gene products that are expressed throughout the nervous and immune systems. These genes encode precursor proteins, from which the active peptide transmitter is subsequently cleaved. Neuropeptide-encoding messenger RNAs (mRNAs) can be found in neuronal processes beyond the perikaryon. SP is synthesized in small- and medium-sized neurons of DRG and stored in dense core vesicles and transported by fast axonal transport to both spinal and peripheral nerve terminals (Hoyer and Bartfai 2012). It binds to tachykinin receptors [neurokinin-1 receptor (NK1R), NK2R, NK3R] that belong, like most neuropeptide receptors, to the family of seven-transmembrane, G protein-coupled receptors. All tachykinins interact with all the three-receptor subtypes with SP preferring NK1, NKA preferring NK2 and NKB (encoded by the TAC3 gene in humans and by the tachykinin 2 (TAC2) gene in rodents) preferring NK3. This lack of specificity can be accounted for by the conformational flexibility of the short, linear peptides (Ganjiwale and Cowsik 2013). Additionally, some of the multiple subtypes and splice variants of these receptors form heterodimers with other neuropeptides and regulate, e.g., trafficking and resensitization of receptors (Pfeiffer et al. 2003). Toxins such as saporin bind to NK receptors (NKRs) and kill, e.g., dorsal horn neurons after they have been internalized following activation (Wiley et al. 2007; Iadarola et al. 2017).

Peripheral nerve injury and inflammation change the phenotype of neurons with regard to receptors and messengers (Weisshaar and Winkelstein 2014). SP released from primary afferent fibers during inflammation upregulates NK1 receptors in dorsal horn neurons. Peptidase inhibitors, which prevent SP breakdown, enhance peptidergic transmission. NKR couples to phospholipase C generating intracellular messengers whose downstream effects include depolarizing the membrane and facilitating the function of α-amino-3-hydroxy-5-methyl-4-isoxazolepropionic acid (AMPA) and NMDA receptors (see below). They, furthermore, control the expression of cytokines and chemokines as well as transcription factors such as nuclear factor kappa-light-chain-enhancer of activated B cells (NF-kB) (Bekhbat et al. 2017) and members of the nuclear hormone family PPAR (Okine et al. 2018). NF-kB is a ubiquitous transcriptional activator of inflammatory mediators that increases the synthesis of pro-inflammatory factors such as cytokines, prostaglandins and nitric oxide that contribute to the development of hyperalgesia (Petho and Reeh 2012).

Whereas NK1 receptors in the hippocampus are downregulated in rat models of pain and stress (Duric and McCarson 2005), they are upregulated in neurons of superficial laminae in the spinal cord (Bradesi et al. 2009). Inflammation and stimulation of nociceptors by capsaicin triggers NKR endocytosis in neurons in superficial laminae of the dorsal horn reflecting sustained release of SP (Kunde et al. 2013). Microglial cell activation plays a major role in the development of this nociceptive sensitization (Wieseler-Frank et al. 2004; Li et al. 2015) (see below). The half-life of the SP response is defined by the kinetics of degradation of the neuropeptide in the extracellular environment and by the dynamics of desensitization and cellular internalization followed by recycling of the receptor. Noteworthy, ligand-induced internalization of NK1 receptors into neurons in the dorsal horn can be triggered also by non-noxious somatosensory stimulation (Honor et al. 1999).

### SP antagonists

Many potent and selective non-peptide, low molecular tachykinin antagonists have been developed and proven effective in preclinical studies (Carvalho et al. 2018). Early experiments showed that antagonists selectively block nociceptive responses such as the slow, prolonged, excitatory postsynaptic potential that follows intense electrical stimuli to small high-threshold multimodal nociceptors (De Koninck and Henry 1991). Unfortunately, most of the knowledge obtained from preclinical studies on nociception has not yet been translated into new therapies. This failure could be due, at least to some extent, to a misconception of what characterizes pain as a chronic disease. Both preclinical and clinical evidence support the involvement of tachykinins and NK1 and NK2 receptors in the neurobiology of depression and anxiety disorders, key features of suffering during chronic pain states (Ebner et al. 2009; Bardelli et al. 2013; Catena-Dell’Osso et al. 2013; Kormos and Gaszner 2013; Rupniak and Kramer 2017).

### Neuromodulation and volume transmission

The discovery of peptidergic neurotransmission in numerous neuronal structures, where the release site and the target do not have to be in close contact, together with synaptic processes by which given neurons use more than one signaling molecule was accompanied by a considerable debate over the definition of a neurotransmitter (Hökfelt et al. 1980; Calza et al. 1998).

The terms neuromodulation and volume transmission were introduced. They describe calcium-dependent signaling at synaptic and non-synaptic sites by neuropeptides co-stored and co-released with classical transmitters. Neuropeptides like SP diffuse farther from their site of release in the intercellular space to reach mostly high-affinity receptors at more remote targets and to regulate synaptic transmission in diverse populations of neurons by volume transmission (Nakamura et al. 2013). This form of chemical communication is much less spatially distinct than classical neurotransmitter signaling and it seems unlikely that neuropeptides participate in rapid integrative functions between nerve cells (Zieglgänsberger 2009).

In a number of primary afferent fibers, SP and other members of the tachykinin family co-exist with glutamate (Hunt and Manthy 2001). Neuromodulation is often contrasted to classical fast synaptic transmission in which an axon terminal secretes neurotransmitters to a restricted target carrying fast-acting receptors and the partner neuron has high affinity reuptake mechanisms that remove the transmitter from the synaptic or extracellular space. Most peptide transmitters lack such a high-affinity reuptake process and are mainly inactivated enzymatically. Such a mechanism markedly extends the signaling capability of neurons in a neuronal network to subsequent stimuli for a period of seconds or even minutes. The actions of certain peptidases may lead to further biologically active fragments making it sometimes difficult to distinguish between extracellular synthetic processing and inactivation. Usually, the peptide that is stored in dense core vesicles and then released is considered the neurotransmitter. Since its release most often requires burst activity or higher frequencies and intensities of stimulation, the signaling of the peptide appears primarily restricted to specific circumstances, i.e., a developing inflammation. The release of fast-acting neurotransmitters, such as glutamate and neuropeptides, may thus contribute significantly to the diversified responses to external stimuli (Zieglgänsberger 2009).

### Cortical and subcortical networks and descending pathways

The role of SP in cortical and subcortical networks as well as in descending pathways involved in the processing of nociceptive inputs is still largely unclear (Budai et al. 2007; Khasabov et al. 2017). Neurons with their perikarya in laminae 1 and 5 project to various sites of the rostral brain such as the caudal ventrolateral medulla, parabrachial area, periaqueductal gray and thalamus as a major relay (Ralston 2005; Todd 2002; Marshall et al. 1996). A recent study underlines the importance of topographic organization of spinofugal connections for nociceptive topognosis, i.e., the ability to localize painful stimuli. Avoidance of environmental nociceptive dangers depends on somatotopic maps arising from topographically organized point-to-point connections between the body surface and the central nervous system (CNS). Humans with an impaired topognosis experience, for example, bilateral sensation evoked by unilateral somatosensory stimulation (da Silva et al. 2018).

Functional magnetic resonance imaging studies have shown that stress exposure reduces pain responses by activation of descending pain inhibitory circuits and may be an indicator of adequate centrally mediated pain control. It has been reported that SP drives endocannabinoid-mediated disinhibition in the periaqueductal/rostral ventromedial medulla projection system, which facilitates these descending pathways to the dorsal horn by an enhancing glutamatergic receptor-mediated function (Drew et al. 2005, 2009). Stress-induced analgesia apparently involves similar sensory, affective and cognitive modulatory brain circuits as placebo analgesia or analgesia mediated by diffuse noxious inhibitory controls (DNICs). The increase in pain tolerance and pain unpleasantness correlated significantly with activation in the rostral anterior cingulate cortex (Yilmaz et al. 2010). Recently, a cingulate cortex/posterior insula pathway was identified that can induce and maintain nociceptive hypersensitivity in the absence of conditioned peripheral noxious drive (Tan et al. 2017).

### Structural and functional changes induced in dendritic spines by noxious stimulation

**Fig. 1:**
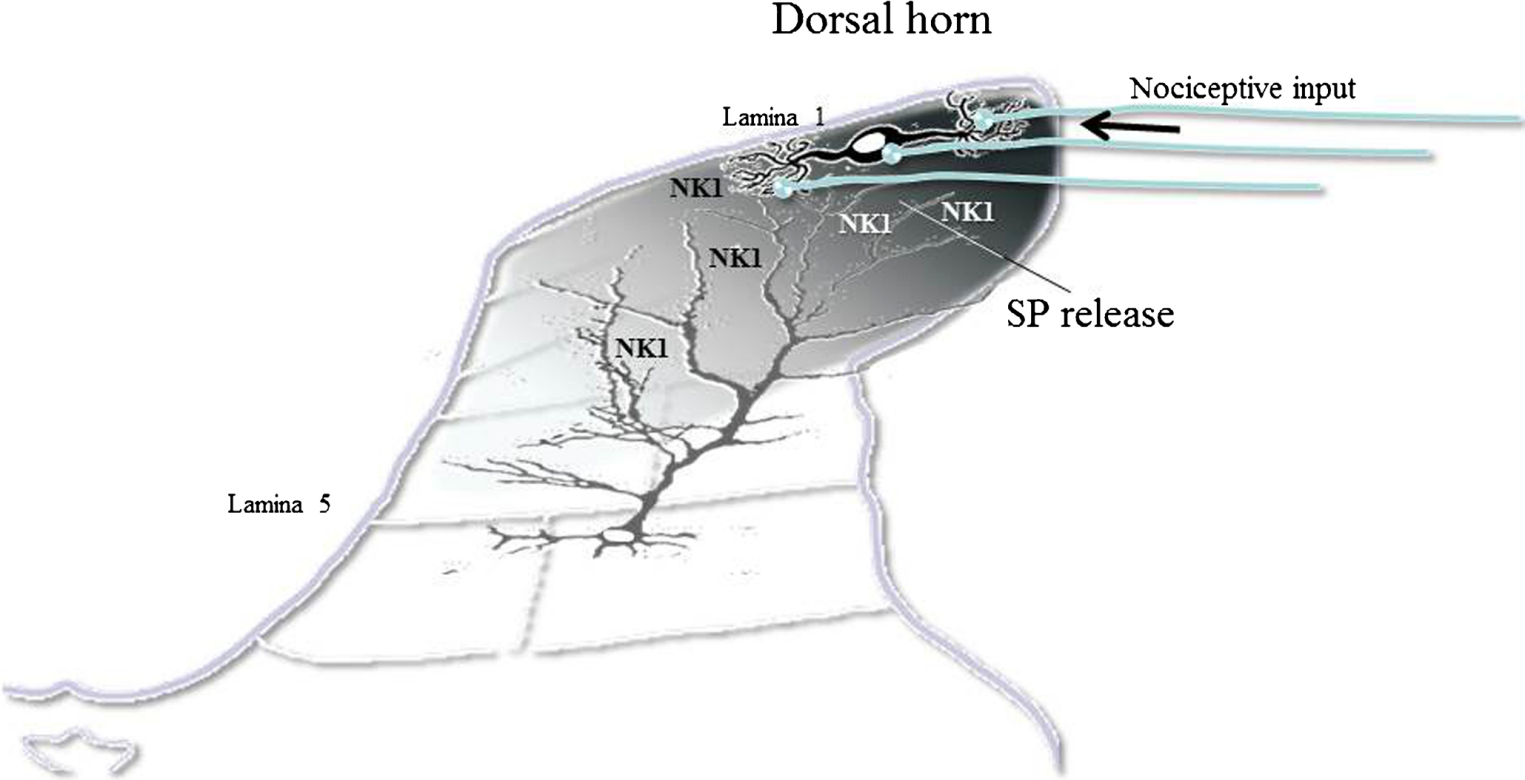
Volume transmission. Sketch illustrating the release of substance P (SP) from primary afferent fibers following nociceptive input. The neuropeptide is released at synaptic and non-synaptic sites in the dorsal horn and reaches neurokinin (NK1) receptors on lamina 1 and NK1 receptors on dendrites of lamina 5 neurons

Experience results in long-lasting changes in dendritic spine morphology, yet how the molecular architecture of the synapse responds to plasticity remains poorly understood. SP-containing terminals of small unmyelinated fibers are localized to the dendritic shafts and spines of spinofugal projection neurons, where they form axodendritic synapses on neurons (Mantyh 2002). However, SP released from primary afferent fibers presynaptically at non-junctional sites could also reach NKR distributed over the length of the long extending dendrites of dorsal horn projection wide dynamic range (WDR) neurons with their soma in lamina 4/5 via volume transmission (see Fig. 1). SP enhances responses to locally applied glutamate in these neurons (Zieglgänsberger and Herz 1971; King et al. 1997, 2005).

Dendritic spines are highly dynamic during development but also in adulthood. They are probably the main targets for SP/glutamate interactions. Following spinal cord injury, the dendrites of WDR neurons located in laminae 4–5 exhibit increased spine density and redistributed spines, compared with controls. Unit recordings in vivo revealed an increase in responsiveness to innocuous and noxious peripheral stimuli under these conditions. These changes were ameliorated by the inhibition of the small GTP-binding protein Rac1 that also increased pain thresholds over a 3-day period (Tan et al. 2008). It is feasible to assume that voltage-gated calcium channels (Nanou and Catterall 2018) trigger this remodeling that contributes to the development of hyperexcitability in these neurons, which is reflected in symptoms of tactile allodynia and thermal hyperalgesia.

### Spine size and shape are controlled by actin dynamics

Stimulated emission depletion (STED) can visualize actin cytoskeleton rearrangements of dendritic spines in living brains. The neuronal filamentous (F)-actin network anchors postsynaptic receptors and modulates synaptic activities, e.g., through the organization of the postsynaptic density (Willig et al. 2014). More recent studies employing novel technologies revealed that the organization and plasticity of spine synapses is linked to the addition of unitary synaptic nanomodules to spines and underlines the important role of NMDA receptors in structural plasticity (Hruska et al. 2018). The calcium-dependent cysteine protease calpain cleaves structural cytoskeleton proteins involved in synaptic plasticity and interferes with memory formation and maintenance in contextual fear conditioning, a process highly relevant for the development of chronic pain states (Popik et al. 2018).

Actin polymerization/depolymerization cycles control structural cytoskeleton proteins and other targets involved in memory formation and maintenance. A murine ortholog of the putative tumor suppressor gene DRR1’s product is a unique stress-induced protein in the brain that binds to actin, promotes bundling and stabilizes actin filaments and impacts actin-dependent neurite outgrowth. Stress has been identified as a major causal factor for many mental disorders and DRR1 emerges as a protein to link stress with actin dynamics related to synaptic function and cognition. Hippocampal virus-mediated enhancement of DRR1 expression reduces spine density, diminishes the probability of synaptic glutamate release and alters cognitive performance (Schmidt et al. 2011).

Individual vulnerability factors influencing the function of the hypothalamic-pituitary-adrenal (HPA) axis contribute to the risk of the development of chronic pain states after traumatic stress exposure (Benedetti et al. 2012; Bortsov et al. 2013). The glucocorticoid receptor co-chaperone FKBP51 acts as an intracellular scaffolding protein and has been associated with depression and post-traumatic stress disorder (Matosin et al. 2018; Fries et al. 2017) and the regulation of stress responses in numerous sites, including the spinal dorsal horn. FKBP51 most likely reduces the severity of persistent pain by modulating glucocorticoid signaling, even though FKBP51 has been found to interact with multiple other proteins in a cell type-specific manner and may thereby affect a variety of biological processes independent of GR signaling (Balsevich et al. 2017a; Gassen et al. 2014). Deletion of spinal FKBP51 after the establishment of inflammation significantly improved the pain state, suggesting that FKBP51 could regulate nociception, independent of its effect on mood. Silencing of FKBP51 alleviates pain by reducing inflammatory factors through the NF-kB signaling pathway (Yu et al. 2017). Blockade of FKBP51 has been suggested as a novel strategy to treat persistent pain (Maiarù et al. 2016). The enhanced mechanical sensitivity of inflamed hind paws accompanied with corticosteroid receptor upregulation in spinal and peripheral sensory neurons was attenuated immediately after glucocorticoid receptor agonist and mineralocorticoid receptor antagonist administration. This suggests acute non-genomic effects consistent with detected membrane-bound corticosteroid receptors (Li et al. 2018). Specific inhibitors of FKBP51 are now available, enabling animal models of long-term pain to be studied (Maiarù et al. 2018). Recent studies show that functional interactions between glucocorticoid signaling and the endocannabinoid system are critical for a wide array of physiological processes (Balsevich et al. 2017b).

## Dorsal horn and nociception

### Primary afferent sensory neurotransmission

Ablating or functionally compromising sets of primary afferent fibers has provided important insights into peripheral modality-specific wiring in the somatosensory system. Nociceptive input into the dorsal horn is not simply passively received. It is modulated by segmental interneurons and descending supraspinal pathways that provide the neuronal circuits by which cognitive and motivational aspects influence the processing of the nociceptive input at this early stage of integration (Eliava et al. 2016). The neuropeptide oxytocin (OT) has profound prosocial effects in non-vertebrate and vertebrate species, including humans (Grinevich et al. 2016). The descending modulatory system can either inhibit or facilitate transmission of nociceptive information. The hypothalamic paraventricular (PVN) and supraoptic (SON) nuclei harbor parvocellular OT cells that project to the brainstem and to neurons of deep layers of the dorsal horn of the spinal cord. Magnocellular OT neurons of these nuclei also innervate numerous forebrain regions and release OT into the blood from the posterior pituitary. There is evidence that the rewarding/reinforcing effects of SP develop by modulating the mesencephalic dopaminergic system, while their mnemonic effects are mediated via the mesencephalic dopaminergic and the basal forebrain cholinergic systems (Lénárd et al. 2018). SP exerts neuromodulatory effects on pain processing and central synaptic transmission in hippocampal areas important for social interaction. SP can induce a slowly developing NMDA receptor- and protein synthesis-dependent potentiation of synaptic transmission and could prime hippocampal synapses for the formation of long-lasting plasticity and associativity (Dasgupta et al. 2017).

**Fig. 2:**
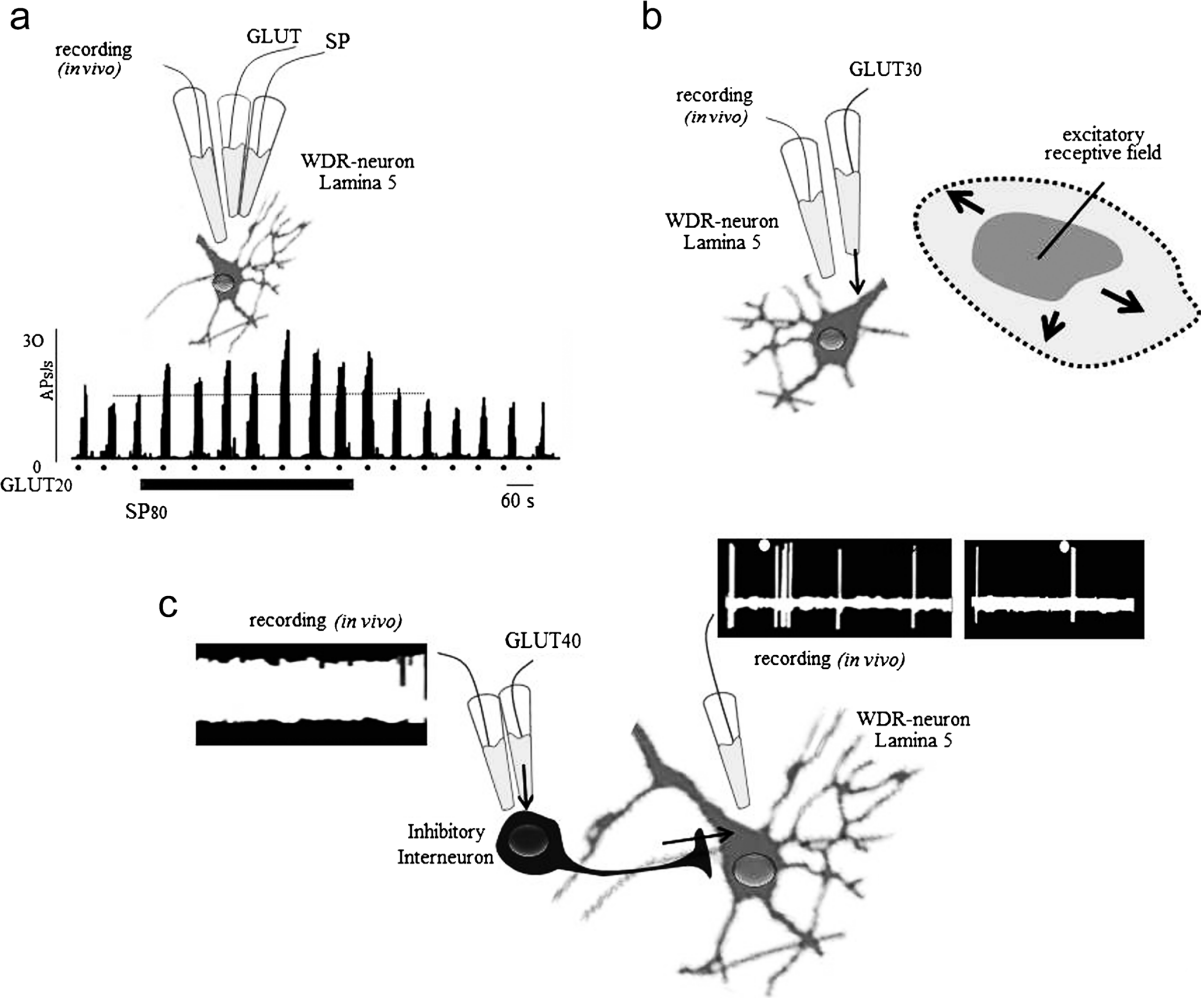
Substance P (SP) increases excitability of glutamate. **a** Microiontophoretically applied SP (80 nA) increases the excitation induced by short applications of l-glutamate (20 nA) in a WDR neuron in lamina 5 recorded extracellularly in vivo. **b** Microiontophoretically applied glutamate transiently increases the size of the receptive field of lamina 5 neurons. The application of GABA shrinks the receptive field (not shown). **c** Activation of an inhibitory interneuron by extracellularly applied glutamate (40 nA) reduces responses to nociceptive stimuli (dots) evoked in a lamina 5 neuron

It has been suggested that the excitability of spinal lamina 1 neurons may play an almost exclusive role in the transmission and processing of nociceptive input leading to persisting pain. However, the release of SP and glutamate lowers the pain threshold of dorsal horn projection neurons originating from lamina 5 WDR neurons and increases the size of their receptive field (Zieglgänsberger and Herz 1971; King et al. 1997; Schadrack and Zieglgänsberger 2000; Martin et al. 2004; Mantyh and Hunt 2004) (Fig. 2). In line with these results, long-term nociception following the injection of Freund’s adjuvant into, e.g., the hind paw of an animal induces the expression of NK1 receptors and transforms non-nociceptive neurons into nociceptive neurons (Almarestani et al. 2009). The enhanced spinofugal output following this alteration of neuronal properties markedly increases the development of chronic persisting pain (see below). Recent studies employing optogenetic tools in freely moving animals show that selective stimulation of non-nociceptive primary afferent Aβ fibers evokes neuropathic pain-like sensory and emotional behaviors after peripheral nerve injury. Notably, these Aβ fiber-mediated responses were observed in lamina 1 neurons and were resistant to morphine. Whole-cell recording and activity markers such as c-Fos and phosphorylated extracellular signal-regulated protein kinase (pERK) revealed excitation of lamina 1 neurons, which were normally silent and activation of central amygdaloid neurons related to aversive behavior (Tashima et al. 2018).

Iontophoretic application of SP amplifies excitatory responses to glutamate agonists in dorsal horn neurons (Fig. 2a) (Zieglgänsberger and Tulloch 1979), an action blocked by an SP antagonist (Sharif et al. 2005). When glutamate was electrophoretically released by short applications from multibarreled glass pipettes, it elicited rapid, short-lasting increases of the discharge activity of projection neurons without markedly enhancing ongoing activity. Interneurons of the spinal dorsal horn are central to somatosensory and nociceptive processing. In an in vivo experiment, exciting segmental interneurons evoked a massive inhibition of the projecting neurons and a reduction of the response to afferent noxious stimuli (Zieglgänsberger and Herz 1971) (Fig. 2c). It has long been appreciated and translated into new therapies that a diminished synaptic inhibition via segmental interneurons in the spinal dorsal horn may be a major contributor to the development of chronic pain states (Zieglgänsberger 1986; Hunt and Mantyh 2001; Mantyh and Hunt 2004; Alles and Smith 2018). A more recent study of the intrinsic properties and the integration of these neurons into dorsal horn circuits showed that tonic firing prevailed in inhibitory interneurons of the dorsal horn. In contrast, delayed firing and single action potential firing were the single most prevalent firing pattern in neurons of the superficial and deep dorsal horn (Punnakkal et al. 2014).

SP activates NKR, which couples to phospholipase C and facilitates glutamatergic transmission by phosphorylation of subunits of the NMDA receptor. Whole-cell patch-clamp recordings in freshly dissociated rat dorsal horn neurons revealed a potentiation of l-glutamate-induced inward currents by SP in a majority of neurons, suggesting a postsynaptic mechanism of action of tachykinins in the rat spinal dorsal horn (Randić et al. 1990). There is evidence that signal transduction coupling between group I metabotropic glutamate receptors and NMDA receptors facilitates the activation of NMDA receptors and may play a critical role in spinal hyperexcitability and hyperalgesia. Inflammation enhanced NMDA receptor-mediated, dorsal root, stimulation-evoked excitatory postsynaptic currents and NMDA-induced currents. The increase in the frequency and amplitude of miniature excitatory postsynaptic currents in the presence of tetrodotoxin suggests an enhanced presynaptic glutamate release probability and postsynaptic membrane responsiveness under these conditions (Yang et al. 2011). In the lamprey, SP depolarizes spinal cord neurons by inhibiting background potassium channels (Thörn Pérez et al. 2015). In DRG neurons, the application of SP evokes a long-lasting increase of an NMDA-activated inward current (Jun et al. 2004). This modulatory effect of SP was blocked by the extracellular application of an SP antagonist (Wu et al. 2004).

### The potassium chloride co-transporter KCC2

**Fig. 3:**
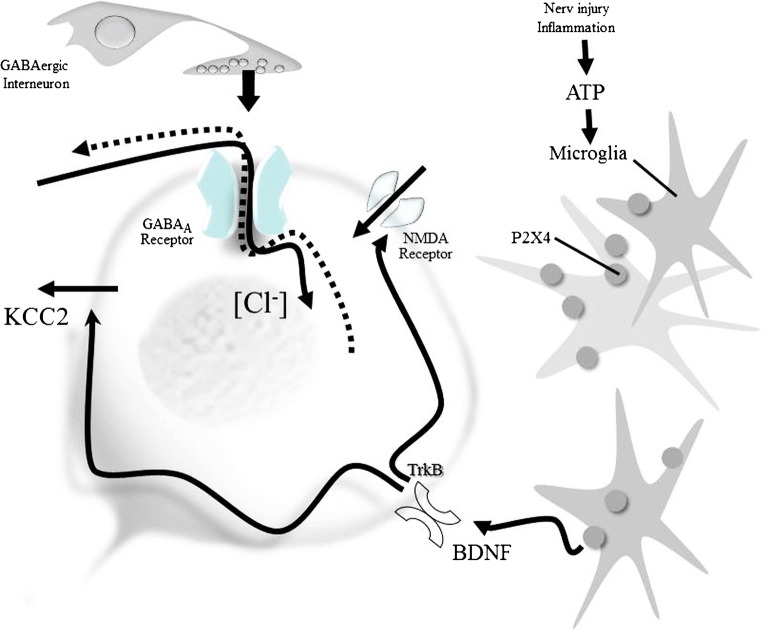
Downregulation of the expression of KCC2 by BDNF released from microglia

γ-Aminobutyric acid (GABA)/glycine-induced neuronal inhibition is associated with a chloride influx that depends on the inwardly directed chloride electrochemical gradient (Fig. 3). Recent studies on loss-of-function mutations in genes encoding glycine receptors and glycine transporters underline the importance of glycinergic neurotransmission for central pain modulation in humans (Vuilleumier et al. 2018). Neuronal intracellular [Cl]^−^ concentration influences membrane potential dynamics and neuronal inhibition in spinal neurons (Javdani et al. 2015). The perikarya and dendrites of dorsal horn neurons of rats widely express the potassium chloride co-transporter KCC2 (Agez et al. 2017), which controls the intracellular [Cl]^−^ concentration. Nerve injury and inflammation downregulate this transporter (Modol et al. 2014). Recent results suggest that the chloride dysregulation may be transient (Castro et al. 2017). A variable density of KCC2 results in variable postsynaptic potentials evoked by GABA_A_ and glycine receptors along the dendrites of these neurons. Most importantly, the shift in the transmembrane anion gradient evokes a loss of inhibition and can invert the inhibitory effect of GABA/glycine released from segmental spinal interneurons into a net excitation (Coull et al. 2003; Price et al. 2005, 2009). Brain-derived neurotrophic factor (BDNF) released from microglia following the activation of ATP-gated purinergic receptors following peripheral nerve injury or inflammation evokes a rapid trans-synaptic reduction in the expression of the KCC2 exporter (Biggs et al. 2010; Ferrini and De Koninck 2013). The intracellular [Cl]^−^ concentration can be restored by novel chloride extrusion enhancers (Gagnon et al. 2013), KCC2 gene transfer (Li et al. 2016), or intrathecal histone deacetylase (HDAC) inhibitor injections, suggesting an effect via epigenetic actions (Lin et al. 2017). Recent studies have shown that P2X4 receptors are upregulated in pain-processing neurons during inflammation (Lalisse et al. 2018). The downregulation of the expression of KCC2 by BDNF released from microglia during acute nociceptive stimulation can be prevented by the application of a kinase inhibitor before stimulation (Tsuruga et al. 2016). In a most recent study, it was shown that mechanical allodynia, a major symptom of neuropathic pain whereby innocuous touch evokes severe pain, cannot be evoked in animals that have peripheral sensory neurons expressing TrkB-positive neurons ablated. The study employing selective optogenetic activation identified a population of TrkB-expressing neurons both necessary and sufficient for producing pain from light touch after nerve injury in mice. The peripheral neurons that transmit pain from light touch might be a novel target for the treatment of neuropathic pain states (Dhandapani et al. 2018).

The paradoxical depolarizing action of GABA/glycine enhances glutamatergic (NMDA) responses by directly activating voltage-sensitive NMDA channels and by BDNF enhancing the phosphorylation of the GluN2B subunit via the Src family kinase Fyn (Hildebrand et al. 2016). Knockdown of KCC2 by intrathecal administration of KCC2-siRNA reduces inhibitory synaptic transmission (Lin et al. 2017). mRNA-mediated downregulation of KCC2 and vesicular GABA transporter expression in the spinal cord contribute to visceral pain in rats (Zhang et al. 2017). Dynamic changes in the mRNA profile revealed multiple regulatory mechanisms in spinofugal projection neurons (von Schack et al. 2011). Unexpectedly, not the injured but adjacent afferents showed the most robust downregulation of miRNA. In uninjured rats, BDNF reduced membrane-bound KCC2 and the inhibitory effect of the GABA_A_ agonist muscimol. After spinal cord injury, BDNF increased KCC2 expression, which might help restore GABAergic inhibition (Huang et al. 2017).

WDR-type response properties were previously almost exclusively observed in neurons located in deeper laminae (Basbaum 1999). The shift of response properties of lamina 1 neurons to a WDR type markedly increases nociceptive signaling to rostral brain structures and enhances lasting maladaptive plasticity in neuronal circuits, e.g., in the limbic system. In monoarthritic animals, the expression of SP and its receptor is increased and the baseline activity of WDR neurons is greater than that in control animals. Disruption of inhibitory control supports long-term potentiation (LTP) in both groups of projection neurons in the dorsal horn and contributes to the development of persisting pain. Previously innocuous stimulation then evokes discharge patterns characteristic for nociceptive stimulation (Pitcher and Henry 2004; Sharif et al. 2005). A synopsis of this data suggests that KCC2 may represent an important novel target for the development of analgesics (Gu et al. 2017a, b).

Prevention of the phosphorylation of the Na-K-Cl co-transporter (NKCC1) avoids downregulation of KCC2 in central sensory pathways and reduces neuropathic pain after peripheral nerve injury (Mòdol et al. 2014). An upregulation of NKCC1 (Price et al. 2009; Glykys et al. 2017) during inflammation produces excessive GABAergic depolarization in primary afferents that leads to cross-excitation between low- and high-threshold afferents (Price et al. 2005). The activation of GABA_A_ receptors on afferent fibers facilitates the development of hyperalgesia induced by inflammation (Lee et al. 2018). The voltage-gated sodium channel Na_v_1.7 that is encoded by the gene *SCN9A* and preferentially located in nociceptors might lose its exclusive role under such conditions. Inflammatory hyperalgesia, cold pain and noxious mechanosensation have all been shown to depend also upon Na_v_1.8-positive sensory neurons. The voltage-gated tetrodotoxin-resistant sodium channels Na_v_1.8 and Na_v_1.9 (coded by *SCN10A* and *SCN11A*, respectively) are also expressed in small-diameter, unmyelinated primary afferents and are currently being investigated for novel pain treatments. Interestingly, Na_v_1.7 blocking spider toxins produced profound analgesia when applied in combination with an opioid agonist at subthreshold concentrations. It was suggested that a lack of Nav1.7 channels may upregulate the expression of the efficacy of antinociceptive opioid signaling mediated by the Gαi-coupled mu opioid receptors (Isensee et al. 2017).

Immune cell recruitment of SP is a critical component of a number of pathologies; e.g., SP recruits leucocytes to the peripheral terminals of nociceptors where they release neuroactive mediators that contribute to neuropathic pain (González-Ramírez et al. 2017; Sousa-Valente and Brain 2018). Human autoantibodies to contactin-associated protein-like 2 (CASPR2) are often associated with neuropathic pain disorder and CASPR2 mutations have been linked to mechanical pain-related hypersensitivity in the absence of neural injury. Mice lacking CASPR2 (Cntnap2−/−) demonstrated enhanced pain-related hypersensitivity to noxious mechanical stimuli, heat and algogens. Both primary afferent excitability and subsequent nociceptive transmission within the dorsal horn were increased in Cntnap2−/− mice. Either immune- or genetic-mediated ablation of CASPR2 enhanced the excitability of DRG neurons in a cell-autonomous fashion through regulation of K_v_1 channel expression at the soma membrane. These data suggest a key role for CASPR2 in regulating cell-intrinsic DRG neuron excitability (Dawes et al. 2018).

Stimulation of unmyelinated C fibers, collaterals, or spinal ganglion cells and even central terminals in the dorsal horn initiate antidromic activity. This activity releases biologically active agents such as SP and calcitonin gene-related peptide (CGRP) and possibly other sensory messengers from distal afferent terminals that evoke local inflammatory responses in the innervated organ systems (Sorkin et al. 2018). These inflammatory processes are referred to as neurogenic inflammation. Recent studies have suggested that electrical coupling via Cx36-containing gap junctions between peripheral sensory fibers containing SP and CGRP exists and that, to some degree of selectivity, electrical coupling may occur between fibers belonging to subpopulations of sensory neurons (Nagy et al. 2018).

## Memory of pain

Actions elicited by SP not only transmit and integrate nociceptive signals but also control their consequences, which are anxiety and depression (Parent et al. 2012). Numerous animal and human studies suggested a role of SP and other members of the tachykinin family in the regulation of biopsychosocial stress responses and emotion processing (de Felipe et al. 1998; Ebner et al. 2009; Madaan and Wilson 2009; Han et al. 2015). A most recent study revealed a profound effect of social isolation stress on the tachykinin system. Enhancing NKB expression and release phenocopied social isolation stress in group-housed mice, promoting aggression and converting stimulus-locked defensive behaviors to persistent responses. The multiple behavioral changes evoked by brain-wide upregulation of TAC2/neurokinin B could be blocked by systemic administration of an NK3R antagonist (Zelikowsky et al. 2018).

The memory of pain is built up by persistent nociceptive, aversive input and should be considered as an experience-based adaption of expectancies and conditioned fear avoidance (Zieglgänsberger et al. 2005). Neurons in the reward system and the amygdala are activated by painful stimuli. Neurons expressing CGRP in the parabrachial nucleus appear critical for relaying nociceptive signals to the central nucleus of the amygdala. After a stable threat memory has been built, a neutral stimulus would then be perceived as being aversive and painful (Han et al. 2015). These neuronal circuits evaluate the expectancy to a known condition and transduce the affective motivational aspects of nociception and thus may be pertinent for treating psychiatric comorbidities (Radat et al. 2013; Vachon et al. 2013).

### Acute vs chronic pain

Pain is one of the most prevalent health conditions in the world. It is widely accepted that only acute pain has an important protective role and warns the organism of an imminent danger, whereas chronic pain usually persists beyond its biological usefulness and compromises the quality of life (Urban and Gebhart 1999; Chang et al. 2010). Most importantly, chronic pain is not simply a temporal continuum of acute pain. In the setting of persistent injury, functional and structural reorganization of neuronal circuits in the CNS leads to long-term changes in perception and behavior (Kuner and Flor 2016).

Associative memory can be inactivated and reactivated by LTP and long-term depression (LTD), respectively, supporting a causal link between these synaptic processes and memory. Although it is clear that chronicity of pain involves learning and psychosocial factors, it remains unclear how they act at the level of neuronal circuits and how the molecular architecture of the synapse responds to plasticity. Compared to the dynamic changes in synaptic connectivity through the growth and elimination of synaptic structures, large-scale organization of axonal and dendritic arbors seems to be rather stable. Rapid developments in technologies to visualize and manipulate circuits, such as infrared microscopy (Dodt et al. 2013), stimulated emission depletion (Willig et al. 2014) and optogenetics (Chen et al. 2018), have opened new avenues for attempts to detail key brain functions and to gain a mechanistic understanding of nociception and pain.

It is well established that the low-frequency afferent barrage during inflammation and trauma enhances pain sensitivity by altering synaptic efficacy (Ikeda et al. 2006) and activity-dependent gene expression in spinal neuronal circuits involved in the transmission and processing of nociceptive signals. The c-Fos immediate early gene has been used as an activation marker for neurons in the superficial dorsal horn (Schadrack et al. 1998, 1999; Schadrack and Zieglgänsberger 2000; Berthele et al. 1999). An increase in synaptic strength involves the de novo formation or elaboration of postsynaptic dendritic structures and constitutes a structural basis for learning and memory in the CNS. LTP plays a key role in the processing and transmission of nociceptive signals to rostral structures via ascending projecting pathways originating from superficial and deep dorsal horn neurons (Rygh et al. 2006). Additionally, it involves specific changes in signaling molecules and intracellular messengers in glia cells (Xanthos and Sandkühler 2014; Kronschläger et al. 2016). The increase in synaptic strength and neuronal excitability includes a cascade of alterations of ionotropic NMDA, AMPA and kainate receptors as well as metabotropic receptors for glutamate (Sheng et al. 2018). Vesicular glutamate transporters sequester glutamate into synaptic vesicles and are reliable markers for glutamatergic neurons and can be used to impair glutamatergic signaling in animal models. They change the temporal and spatial profile of a neurotransmitter and can affect the downstream effects of these neurotransmitters and hence modulate pain (Yousuf and Kerr 2016).

Since many forms of synaptic plasticity rely on glutamate, the major excitatory small synaptic vesicle transmitter, used by most, if not all, primary sensory afferents involved in pain signaling, it is not surprising that SP enhances the generation of LTP. SP has to be increased extracellularly during the induction of LTP in these projection neurons. Whereas the maintenance of spinal LTP following high-frequency peripheral nerve stimulation does not depend on increased SP release (Afrah et al. 2002). Also, the enhancement of SP signaling in the motor cortex seems to facilitate learning of a motor task preferentially in the early phase of skill acquisition (Hertler et al. 2017).

### Long-term storage of memory and the perineuronal net

In adult animals, fear conditioning induces memory that appears resilient to erasure. Fear memories may actively be protected in pain-recipient neurons of the amygdala by perineuronal nets (PNNs) (Gogolla et al. 2009). Enzymatic digestion of PNNs by chondroitinase ABC lyase (chABC) in the amygdala renders subsequently acquired fear memories susceptible to erasure. It was suggested that very long-term memories may even be stored in the pattern of holes in a web-like structure of proteins such as brevican, aggrecan, neurocan and hyaluronan cross-linked by link proteins and tenascin-R (Tsien 2013). These specialized reticular extracellular matrix proteins envelop mature neurons and apparently restrict synapse formation and squelch neuronal signaling. In the cortex and other subcortical areas, PNNs preferentially surround GABAergic interneurons containing the calcium-binding protein parvalbumin. These specific cells have been recently identified as fast-spiking cells relevant for gamma-oscillations (Dine et al. 2016).

Cell surface proteins, including neurotransmitter receptors, are highly mobile in the plasma membrane due to lateral diffusion. Fast trafficking of AMPA-type glutamate receptors (AMPARs) is involved in the modulation of synaptic transmission. PNNs compartmentalize the neuronal surface and may act as lateral diffusion barriers for AMPARs and limit synaptic plasticity once PNNs become upregulated (Sheng et al. 2018). Heterodimers formed by NKRs with other neuropeptide receptors control these trafficking and resensitization processes (Pfeiffer et al. 2003).

The enzymatic degradation of PNNs by chABC collapses much of the net-associated extracellular matrix (Brückner et al. 1998; Pizzorusso et al. 2002) and leads to reactivation of plasticity (Pizzorusso et al. 2002; Corvetti and Rossi 2005). chABC applied to lesion-denervated targets has been shown to enhance collateral sprouting and synapse formation by spared fibers (Tropea et al. 2003). The PNN is initially laid down at the end of the critical period in wiring of sensory inputs, a process that may have both neuronal and glial contributions. After the critical period passes, the PNN is quickly produced, which stabilizes the whole neural network, making it difficult for neurons to make new connections (Massey et al. 2006). Inhibition of HDAC can reopen critical-period neuroplasticity in adult mice. HDAC may act as an epigenetic “brake” on critical learning (Nabel and Morishita 2013). The antidepressant fluoxetine increases synaptic plasticity and helps to remodel the fear memory circuitry in the limbic system and apparently converts the fear memory circuitry to a more immature state (Karpova et al. 2011).

Nociceptive inputs create different levels of stress and anxiety depending on their nature, duration and intensity; therefore, the memory of pain can become more damaging than its initial experience. However, not all individuals develop the same level of psychopathology after stress exposure (Santarelli et al. 2017). Depending on the set and setting, chronic and repeated stress can lead to either a reduction or exacerbation of the pain state. Repeated stress can produce maladaptive neurobiological changes in pathways associated with pain processing, resulting in stress-induced hyperalgesia (Jennings et al. 2014). The influence of genetic background on this stress-induced hyperalgesia is poorly understood.

Repeated exposure to stress such as forced swimming alters endocannabinoid signaling differently in the dorsal horn and the amygdala in genetically different animals. These data implicate the endocannabinoid system in the spinal cord and amygdala in these differences (Jennings et al. 2016). It has been shown previously that the endocannabinoid system facilitates the erasure of aversive memory in the amygdala by affecting associative plasticity in inhibitory circuits (Marsicano et al. 2002; Azad et al. 2003). The endocannabinoid system is involved in neuroprotection (Marsicano et al. 2003) and controls key epileptogenic circuits in the hippocampus (Monory et al. 2006).

#### Relearning

Since most neuronal circuits remain plastic throughout life, it should be possible to modify dysfunctional cognition by promnesic drugs and thus increase the patient’s hope for therapeutic relief. Even a reopening of the critical period might be considered. However, superior memory could have a downside and trying to improve memory in humans may provide unintended traits. It has been shown that mice overexpressing the NMDA receptor subunit NR2B are “smarter” but more sensitive to the development of persisting pain, although they reacted like controls in acute pain tests (Tang et al. 1999). Patients often turn to medical cannabis to manage chronic pain states. We still do not have a consolidated perspective as to whether CB1 agonists are drugs of abuse or medicine. At present, there is still a lack of adequate clinical studies in this area. Cannabis is not just another analgesic; rather, it helps relieve the anxiety triggered by the fear of pain, the hallmark of the memory of pain. Recent research suggests that activating the endocannabinoid system ameliorates inflammatory, nociceptive and neuropathic pain and stress and thus may provide a novel treatment option to beneficially influence relearning by alleviating anxiety and depression, the key features of suffering during chronic pain states.

## Outlook

The various members of the tachykinin family are processed differently and bind to distinctive NKR, illustrating the bewildering complexity of this peptidergic system (Hallberg 2015). Many of the fragments generated have not yet been examined in detail with regard to their potential biological activities. The multiple mechanisms involved in these processes may offer unique and important targets for the development of therapeutically relevant agonists and antagonists. SP is also expressed by a variety of non-neuronal cell types such as microglia, as well as immune cells such as T cells, tissue-resident macrophages, dendritic cells and eosinophils. SP is known to play a key role in immune cell migration and expression of chemokines and adhesion molecules (Mashaghi et al. 2016). Factors released from neuronal and non-neuronal elements have been recognized as mediators of persisting pain (Guo et al. 2007; Xanthos and Sandkühler 2014; Totsch and Sorge 2017). T cells may contribute to neuropathic pain that arises from an injury or disease of the somatosensory nervous system through pro-inflammatory interactions with microglia and by facilitating the activation of astrocytes, rather than through nociceptor activation (Grace et al. 2011, 2014). The phenotypic changes observed in transgenic animals lacking components of the neurokinin system are not as dramatic as pharmacological and morphological studies had suggested. This is possibly due to compensatory mechanisms in the tachykinin system.

We witnessed a large drive to identify molecular mediators of pain-related functional plasticity leading to chronicity. Chronic pain is no longer considered an extension of acute pain: it is the result of the memory of pain experienced over time and should be considered as a disease state of the nervous system. Currently, the treatment of chronic pain is inadequate and compromised by debilitating CNS side effects. It is the set and setting of the experience of nociceptive stimuli, rather than their intensity, which makes them memorable. The influence of emotional and cognitive input and feedback from different brain areas makes nociception not only a perception but also an experience. Maladaptive experience-based adaption of expectancies contributes to contextual fear conditioning. The limbic system stores memories of aversive stimuli with no simple mechanism for erasing them. The memory of pain is best countered by overlaying it with positive new associations. Numerous therapeutic algorithms and pharmacological agents have been described to block the generation of nociceptive hypersensitivity in animal models. These steps clearly indicate major progress. However, despite these important advances, their clinical translation remains stunted. Nevertheless, we begin to piece together intricate pathways in the neurobiology of nociception and mechanisms of cell-to-cell communication in modulating nociception leading to pain and suffering.
